# Improving the provision of OTC medication information in community pharmacies in Poland

**DOI:** 10.1007/s11096-016-0397-z

**Published:** 2016-11-30

**Authors:** Anna Piecuch, Magdalena Makarewicz-Wujec, Małgorzata Kozłowska-Wojciechowska

**Affiliations:** Department of Clinical Pharmacy and Pharmaceutical Care, Faculty of Pharmacy, Medical University of Warsaw, ul. Banacha 1, 02-097 Warsaw, Poland

**Keywords:** Community pharmacy, Medicine information, Patient, Pharmacy services, Poland

## Abstract

*Background* An informed or shared decision-making model is desirable to support the choice of over-the-counter (OTC) medications in pharmacies: it respects patient empowerment in self-medication. Such a model is achievable provided that pharmacists are a credible, competent information source open to patient needs. *Objective* To study the dependencies among selected factors that may influence the provision of OTC medication information. The study was conducted from the perspective of a community pharmacist. *Method* The study consisted of an auditorium survey with a self-administered questionnaire. We attempted to determine the relationships among three selected constructs: patient centredness (four items), competence (four items), and provision of OTC medication information (six items) as latent variables. We analysed hypothetical relationships among the observable variables and latent variables using structural equation modelling. *Main outcome measure* Selected factors that may influence the provision of OTC medication information. *Results* In all, 1496 pharmacists took part in the study. The model demonstrated adequate fit (χ^2^ = 198.39, *df* = 64). The patient-centredness construct was demonstrated to have a strong direct positive impact on the provision of OTC medication information construct (β = 0.77, *P* < 0.05). Provision of OTC medication information was also shown to have a strong direct effect on the competence variable (β = 0.90, *P* < 0.05). *Conclusion* If a pharmacist is patient centred, there is a greater possibility that they will provide information about OTC medicines; that may influence the pharmacist’s feelings about their ability to cope with patient initiatives and enhance the pharmacist’s selfperceived competence.

## Impact of findings on practice

- The patient-centred approach should be implemented and supported in pharmacy practice in Poland.
- Poland’s pharmacists should develop their professional competencies for providing information on OTC medications.
- The Polish Pharmaceutical Chamber should support the provision of OTC medication information by issuing appropriate guidelines and advocating legal changes.

## Introduction

An informed or shared decision-making model is desirable when choosing over-the-counter (OTC) medication in a pharmacy [1]: such a model respects patient empowerment in self-medication [2] and results from patient-centred care [3]. The concept of shared decision making is based on information exchange between a specialist (here, a pharmacist) and a patient; it also involves mutual expression of preferences and involvement of both the specialist and patient in the decision-making process [4]. With OTC medications, the final decision about medication choice rests with the patient, who is supported by the pharmacist in responsible self-medication [5]. To make informed decisions about medications, the patient needs an adequate range of clear information about both the risks and benefits of a given product [2, 6]. The range of information and manner of its communication by the pharmacist should result in greater awareness and ability on the part of the patient to help them make informed choices [7]; such information should also ensure that the patient can obtain the greatest benefit by using a given medication [8] and avoid medication problems [7, 9].

To support a patient in meeting medication needs, a pharmacist needs to be credible and competent; this demands expertise and skilful information sharing with the patient (which is part of the shared decision-making model [4]) as well as respecting the patient’s needs [10] (which results from patient-centred care [3]). Having trust in the pharmacist may be understood as, for example, perceiving them to be a reliable source of information. Demonstrating thorough interest in the patient may result in the patient openly expressing their needs and problems as well as asking pertinent questions [10].

The range and quality of information about dispensed medicines during an encounter may depend on strict laws and regulations [11]. In Poland, OTC medications are available at pharmacies [12], and they can be sold without prior interview with or assessment by the patient. OTC medications are also available at dispensaries [13] in rural areas that lack community pharmacies [14]. In addition, some OTC medications may be sold in other retail outlets [15], e.g. general stores. Legal requirements oblige people who dispense medications in Polish pharmacies to provide, if necessary, patients with information about given medications—both prescription and OTC medications. Such requirements particularly apply to the methods of administration, storage conditions, pharmacological effects, and possible interactions with other medications [16].

In practice, however, fulfilling this obligation markedly differs among individual pharmacies; this is due to generalized, ambiguous legal requirements that are particularly questionable concerning the interpretation of the “if necessary” wording. The kind of information supplied is commonly limited to instructing the patient [17]. Interactions between pharmacists and patients in Poland tend to brief and mainly product oriented, not patient centred. Depending on the type of transaction, the content of communication between a pharmacist and patient, including the range of information provided by the pharmacist to the patient, is determined by both participants in the interaction [18]. If the pharmacist waits for the patient to make the initiative and start asking questions, the pharmacist may fail and the patient will not obtain important information [19]. The most important information about a medicinal product is provided in the package leaflet [20, 21]. However, such written information should not replace the communication between a pharmacist and patient but supplement it [22]. Verbal information provided to patients should strictly correspond to their needs [23]. It is known that an adequate interview and assessment are necessary to provide appropriate advice or medical referral; however, there are no commonly accepted rules in Poland concerning the patient interview at a pharmacy [24].

A pharmacist in Poland can influence the choice and use of OTC medications by a patient. The focus of the present study is self-medication.

## Aim of the study

The objective of this study was to explore the interplay between selected constructs (pharmacist’s patient-centredness and competence) that may influence the provision of medication information by pharmacists. The study was conducted from the perspective of a community pharmacist.

## Ethics approval

Ethics approval was not required for this study. According to Polish regulations, non-interventional studies do not require ethical approval [25].

## Method

### Sample

We distributed a self-administered questionnaire among 4537 pharmacists, between 3 March and 24 November 2012. No incentives were offered to participants.

The sample covered pharmacists who took part in an ongoing education course in geriatric pharmaceutical care, delivered by the Centre of Postgraduate Training of the Medical University of Warsaw, Faculty of Pharmacy. The course was directed at pharmacists working at pharmacies, dispensaries, and pharmaceutical wholesalers. The course was a 1-day symposium. One of the lectures addressed the potential for collaboration between a pharmacy and a patient as well as selected issues concerning OTC medicines and self-medication. The course was free of charge and open to all eligible pharmacists who had made a prior registration. The course was delivered according to the same curriculum and by the same lecturers in 16 cities across Poland. Continuing education of pharmacists is compulsory in Poland, and this particular symposium was very well attended by community pharmacists. The course was not held in the area covered by four regional pharmaceutical chambers: Środkowopomorska, Kaliska, Beskidzka, and Częstochowska regional pharmaceutical chambers, which account for 20% of all such chambers in the country.

The inclusion criteria for study participants were as follows: licensed pharmacists working at community pharmacies; licensed pharmacists working at dispensaries. The exclusion criteria were as follows: licensed pharmacists working at hospital pharmacies; licensed pharmacists working at pharmaceutical wholesalers; non-pharmacists, including owners of pharmacies who did not hold a master’s degree in pharmacy, students of pharmacy, and pharmacy technicians; and incomplete data (missing responses to over three items in the 14-item scale).

### Survey

The study questionnaire consisted of three parts. The first part included questions about the relationship between pharmacists and physician and will be discussed in more detail in a subsequent report. The second part addressed pharmacist-patient communication. The third part covered socio-demographic questions.

With the survey, we attempted to explore the interplay among three selected constructs. Discussions within the research team resulted in the development of the initial constructs and indicators. The indicators were then further developed based on in-depth interviews with a convenience sample of eight pharmacists. One of the researchers took notes of the pharmacists’ opinions. The pharmacists talked about the kind of information they provided and, in their opinion, should be provided to patients when selling OTC medications. The pharmacists also spoke about when patients could ask them for help, how they demonstrated their interest in the patient’s welfare, and how they dealt with their own credibility. The patient-centredness construct (PAT) consisted of four items (with one reverse-coded item); the competence construct (COM) was also made up of four items; and the provision of medication information construct (INF) was based on six items (with one reverse-coded item). We applied a five-point Likert scale in the survey, the total score being the sum of all item scores. A respondent had to answer at least 80% of the items. Missing values for one to three items were replaced with the mean score calculated from items completed by the respondent.

An additional question addressed general self-assessment concerning relations between the pharmacist and patient (using a five-point scale from “very good” to “very bad”).

*Patient-centredness*. In this study, PAT was considered in terms of taking care of the patient’s needs as well as acting with good intention and in the patient’s best interest.

*Competence*. COM was defined as the pharmacist’s expertise in OTC medications and their openness to medication needs and patient questions.

*Provision of medication information*. INF was understood as providing patients with basic information necessary to ensure safety and efficacy with self-medication (i.e. method of administration, storage conditions, contraindications, possible side effects, and interactions).

### Analysis

We used Statistica 10 software for statistical analysis. Structural equation modelling using LISREL 8.80 was performed by an external service provider.

## Results

Of 1722 completed questionnaires (response rate, 38.0%), 226 were rejected as incomplete or failed to comply with the inclusion criteria. Thus, 1496 responses qualified for further analysis, accounting for 33.0% of the originally distributed questionnaires. A summary of the socio-demographic characteristics of participants is provided in Table 1. With reference to data from Poland’s Central Statistical Office [26], the proportion of female respondents was slightly higher than in the general population of pharmacists (88 vs. 84%). We found no statistically significant differentiation with regard to socio-demographic factors (*P* < 0.05).

**Table 1:** Sociodemographic characteristics of the study sample

Independent variables	n = 1496 (%)
Age	40 ± 11 years
Sex	
Female	1312 (87.7)
Male	184 (12.3)
Years in practice	
≤5	445 (30.1)
6–10	201 (13.6)
11–15	202 (13.6)
16–20	219 (14.8)
>20	413 (27.9)
Job position^a^	
Pharmacist (with a Masters degree in pharmacy)	1496 (100.0)
Pharmacy manager	630 (42.1)
Pharmacy owner	156 (10.4)
Other job position	2 (<0.1)
Pharmacy setting	
Rural area	115 (7.7)
Urban area of up to 20,000 inhabitants	279 (18.6)
Urban area of 20,000–100,000 inhabitants	365 (24.4)
Urban area of 100,000–500 000 inhabitants	291 (19.5)
Urban area of over 500,000 inhabitants	431 (28.8)
No response	15 (1.0)
Pharmacy	
Independent pharmacy	913 (61.0)
Chain pharmacy	500 (33.4)
Other type of pharmacy	83 (5.5)
Self-perceived pharmacist-patient relationships	
Very good	486 (32.5)
Good	963 (64.4)
Neither good nor bad	44 (2.9)
Bad	3 (0.2)
Very bad	0 (<0.1)

^a^Answers do not sum up to 100% as multiple answers were possible

The median (interquartile range) numbers and proportions of responses to the study items are listed in Table 2. Estimation of the parameters and the fit of the structural equation model indicated good fit of the data to the proposed model. The Chi square test to test the absence of a perfect data-model fit hypothesis was statistically significant: χ (64) = 198.39, *P* < 0.001). The model actually fitted the data very well, as evidenced in the root mean square error of approximation (RMSEA) value of 0.038. It was evident that the latent variables introduced into the model were strongly positively intercorrelated. All the relationships between the observable and latent variables were statistically significant; if they were eliminated, the fit of the model would be negatively affected.

**Table 2:** Descriptive statistics of study constructs—patient-centredness, competence and provision of OTC medication information (*n* = 1496)

Construct/Item	n	Median [IQR]	Strongly agree	Agree	Undecided	Disagree	Strongly disagree	No response
Patient-centredness (reverse coded)								
PAT1. I consider patient well-being as the highest priority	1494	5 [5–5]	1197 (80%)	282 (19%)	12 (<1%)	2 (<1%)	1 (<1%)	2 (<1%)
PAT2. When asked for advice, I recommend a drug which best suits patient needs	1490	5 [5–5]	1186 (79%)	287 (19%)	13 (<1%)	3 (<1%)	1 (<1%)	6 (<1%)
PAT3. I would never recommend a drug that would not work in the patient	1491	5 [4–5]	1105 (74%)	279 (19%)	79 (5%)	24 (2%)	4 (<1%)	5 (<1%)
PAT4. I try to sell drugs with which I can earn the highest profit margin^a^	1481	4 [4–5]	32 (2%)	114 (8%)	208 (14%)	459 (31%)	668 (45%)	15 (1%)
Competence								
COM1. If in doubt as to how a medication works or should be used, patients can always seek my advice	1494	5 [5–5]	1327 (89%)	155 (10%)	8 (<1%)	2 (<1%)	2 (<1%)	2 (<1%)
COM2. If they want to buy an OTC medication, patients should first seek my advice	1490	5 [4–5]	995 (67%)	329 (22%)	72 (5%)	51 (3%)	43 (3%)	6 (<1%)
COM3. I have extensive expertise in medications	1489	4 [4–5]	522 (35%)	753 (50%)	208 (14%)	6 (<1%)	0 (<1%)	7 (<1%)
COM4. I am usually able to comprehensively answer patients’ questions about medications	1481	4 [4–5]	535 (36%)	811 (54%)	126 (8%)	8 (<1%)	1 (<1%)	15 (1%)
Provision of OTC medication information (reverse coded)								
INF1. I always instruct patients on how to correctly use the drug I sell	1491	5 [4–5]	767 (51%)	584 (39%)	90 (6%)	44 (3%)	6 (<1%)	5 (<1%)
INF2. I warn patients of possible adverse drug reactions	1489	4 [3–5]	398 (27%)	587 (39%)	283 (19%)	189 (13%)	32 (2%)	7 (<1%)
INF3. I typically do not ask any additional questions when I dispense medications^a^	1487	4 [4–5]	30 (2%)	118 (8%)	194 (%)	607 (41%)	538 (36%)	9 (<1%)
INF4. I warn patients of possible interactions when I dispense medications	1490	4 [3–4]	290 (19%)	568 (38%)	370 (25%)	211 (14%)	51 (3%)	6 (<1%)
INF5. I always make sure that there are no contraindications for the patient to use the medication	1485	4 [4–5]	272 (18%)	544 (36%)	386 (26%)	220 (15%)	63 (4%)	11 (<1%)
INF6. I typically instruct patients on how to store the drugs I dispense	1488	5 [4–5]	1022 (68%)	372 (25%)	52 (3%)	31 (2%)	11 (<1%)	8 (<1%)

*IQR* Interquartile range

^a^Reverse coded

We calculated skewness and kurtosis for the observable variables. Since the variants did not meet the consistency condition with a normal distribution, we used a weighted least-squares estimator. Spearman’s rank correlation matrix analysis revealed no collinearity among the analysed variables. All the correlations were statistically significant (*P* < 0.05).

The close-fit hypothesis was confirmed and accepted for the designed model [27]: RMSEA = 0.038 was within the range of (0.0; 0.5) for good-fit acceptance; the 90% confidence interval for the RMSEA population value (0.032; 0.044) was within the range of (0.0; 0.5) for good-fit acceptance; and the *P* value for the close-fit hypothesis (RMSEA < 0.05) equalled 1.

**Fig. 1:**
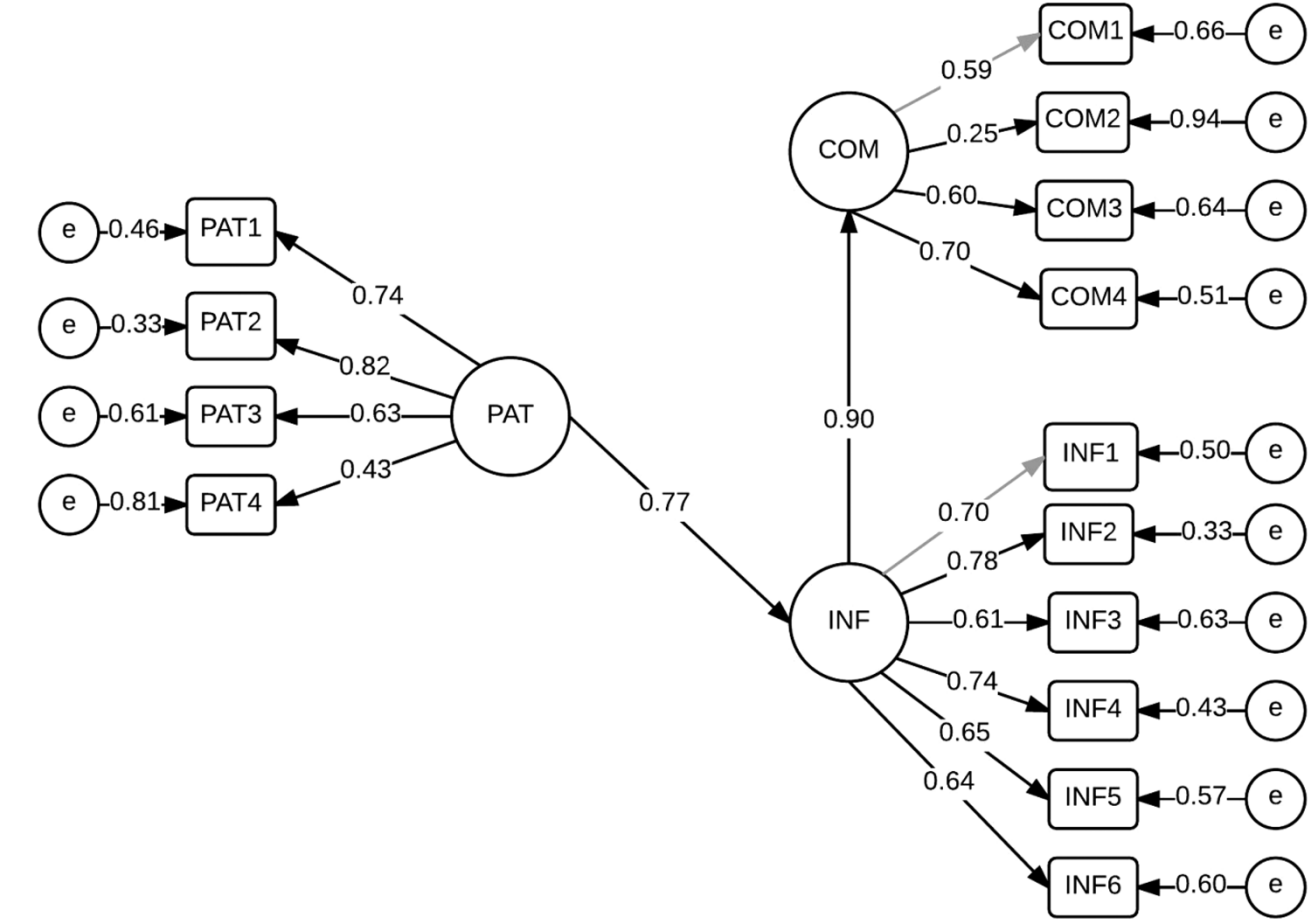
Structural equation model showing relationships among the study constructs: patient centredness, competence, and provision of OTC medication information. χ^2^ = 198.39, *df* = 64, *P* < 0.001, root mean square of approximation = 0.038 COM—competence INF—provision of OTC medication information PAT—patient centredness

In the structural submodel, PAT was not a dependent variable. PAT had a strong direct positive impact on INF (β = 0.77, *P* < 0.05). PAT was also shown to have a strong direct effect on COM (β = 0.90, *P* < 0.05), which means that PAT had an indirect impact on COM by means of INF (Fig. 1). Table 3 presents the results of confirmative factor analysis of the model.

**Table 3:** Confirmative factor analysis of the individual items representing study constructs

Variable	SRW	URW	SE URW	Cronbach coefficient alpha	AVE (%)
Patient-centredness					
PAT1	0.74	2.22	0.11	0.50	45
PAT2	0.82	2.46	0.11		
PAT3	0.63	0.83	0.04		
PAT4	0.43	0.58	0.04		
Competence					
COM1	0.59	4.65	0	0.47	22
COM2	0.25	0.71	0.10		
COM3	0.60	0.37	0.03		
COM4	0.70	1.07	0.08		
Provision of OTC medication information					
INF1	0.70	0.89	0	0.78	37
INF2	0.78	0.80	0.03		
INF3	0.61	0.81	0.04		
INF4	0.74	0.83	0.03		
INF5	0.65	0.76	0.03		
INF6	0.64	1.19	0.05		

*SRC* Standardized regression coefficient, *URW* unstandardized regression weight, *SE* standard error, *AVE* average variance extracted

## Discussion

The patient-centred pharmacist can facilitate self-medication by providing information about OTC medications, dealing with patients’ questions and concerns, and being open to patients’ needs [28]. Many factors can influence the provision of information to patients at a pharmacy: whether any information is provided, the kind of information, its range, and its manner of presentation [29]. The provision of information depends on the pharmacist but also on the patients themselves [30], their needs related to the type of medication being dispensed, their health problems [31], and various external factors. With the final item, the following factors may be of importance: legal conditions and applicable guidelines [17]; and the system or organization of pharmacy activities [32]. In this paper, we examined only two selected constructs that may have an effect on the provision of information related to OTC medication. Owing to differences in the factors that can influence the provision of information to patients, our findings may not apply to other countries.

The analysis of the model showed that pharmacists who consider the position of the patient are more involved in providing OTC medication information; this in turn helps consolidate the pharmacist’s self-perceived competence. In Poland, patients generally base their OTC drug choices on past experience; however, the possibility of consultation with a pharmacist is important for those who select a pharmacy when making an OTC purchase [33]. Previous studies on patient preferences in Poland have demonstrated that a pharmacy’s location as well as the price and availability of drugs are considered more important than the possibility of consultation with a pharmacist [34]; around 50% of Poles use OTC medication for the first time without consulting a physician or pharmacist [35]. These results can be accounted for by the passive behaviour of pharmacists [36], the limited perceived reliability of pharmacist advice regarding medications, and the lack of confidentiality in a pharmacy setting [34, 36, 37]. In the present study, the overall score of PAT was reduced by pharmacists who responded that they would intentionally recommend a drug that would bring them a higher profit; this indicates that the social fear of mercantilism among pharmacies is not entirely unfounded. However, the character of the pharmacist plays a primary role as to whether they are regarded as an ordinary salesman or a trustworthy consultant [38].

The pharmacists rated their competence and ethical conduct relatively highly. However, the responses of some pharmacists suggest that they may not provide comprehensive information to patients who buy OTC medications. The results of this study would appear to be consistent with those of investigations that have indicated that pharmacists in Poland do not always provide adequate, complete information to patients, especially when unsolicited [36, 37]. This may be explained by the fact that pharmacists simply lack the necessary practical skills and self-assurance. Another explanation is that COM was affected by lower ratings of self-perceived expertise in drugs and the ability to respond to patient questions. A further possible issue is that owing to legal requirements, pharmacists were not sufficiently motivated to properly support patient self-medication.

### Limitations

This study consisted of an auditorium survey with a self-administered questionnaire, which had some inherent limitations [39]. The questionnaire was self-administered, but the respondents were able to see and even communicate with one another.

Measurement error was another possible limitation. The main probable cause was the data collection method, in which respondents self-reported their beliefs and behaviours. The study results could have been exposed to an error attributed to social expectations. Respondents may have been compelled to respond in a socially desirable manner rather than truthfully. With a relatively low response rate, error attributed to non-responses cannot be excluded. Moreover, it should be noted that the study covered a period of 9 months; during that time, some factors affecting the studied relationships could have changed considerably.

The pharmacist perspective was adopted in this study. Future research should also compare how patients respond to the same questions.

## Conclusion

If a pharmacist is patient centred and considers the patient’s welfare, there is a greater possibility that they will provide information about OTC medicines; that may influence the pharmacist’s feeling about their ability to cope with patients’ initiatives and enhance the pharmacist’s self-perceived competence.
